# Exploiting the Oral Microbiome to Prevent Tooth Decay: Has Evolution Already Provided the Best Tools?

**DOI:** 10.3389/fmicb.2018.03323

**Published:** 2019-01-11

**Authors:** Jonathon L. Baker, Anna Edlund

**Affiliations:** Genomic Medicine Group, J. Craig Venter Institute, La Jolla, CA, United States

**Keywords:** oral probiotics, oral treatment, *Streptococcus*, caries, antimicrobial small molecules

## Abstract

To compete in the relatively exposed oral cavity, resident microbes must avoid being replaced by newcomers. This selective constraint, coupled with pressure on the host to cultivate a beneficial microbiome, has rendered a commensal oral microbiota that displays colonization resistance, protecting the human host from invasive species, including pathogens. Rapid increases in carbohydrate consumption have disrupted the evolved homeostasis between the oral microbiota and dental health, reflected by the high prevalence of dental caries. Development of novel modalities to prevent caries has been the subject of a breadth of research. This mini review provides highlights of these endeavors and discusses the rationale and pitfalls behind the major avenues of approach. Despite efficacy, fluoride and other broad-spectrum interventions are unlikely to further reduce the incidence of dental caries. The most promising methodologies in development are those that exploit the exclusive nature of the healthy oral microbiome. Probiotics derived from the dental plaque of healthy individuals sharply antagonize cariogenic species, such as *Streptococcus mutans.* Meanwhile, targeted antimicrobials allow for the killing of specific pathogens, allowing reestablishment of a healthy microbiome, presumably with its protective effects. The oral microbiota manufactures a massive array of small molecules, some of which are correlated with health and are likely to antagonize pathogens. The prohibitive cost associated with sufficiently rigorous clinical trials, and the status of dental caries as a non-life-threatening condition will likely continue to impede the advancement of new therapeutics to market. Nevertheless, there is room for optimism, as it appears evolution may have already provided the best tools.

## Introduction

Evolution within a microbiota is driven by the requirement of each taxa to compete and persist within the host. Meanwhile, hosts are under strong selective pressure to modulate their microbiota to ensure that it confers a benefit. Unlike individual microbes, the sophisticated immune system of mammals can easily influence an entire resident microbial community, and benefit from doing so. For this reason, the human microbiota has been described as an “ecosystem on a leash” (Foster et al., 2017). With multiple microenvironments allowing for a large diversity of taxa, as well as consistent exposure to the external environment and food, the oral cavity presents a highly unique circumstance for the interaction of the human microbiota and the host. Constant exposure to foreign microbes selects for oral taxa which are particularly skilled at direct competition—they must avoid being replaced! As a consequence, the oral microbiome displays colonization resistance, which is beneficial to the host (He et al., 2014). Therefore, it is probable that the immunology of the oral cavity has also evolved to tolerate, and even facilitate, maintenance of a commensal, yet fiercely territorial, oral microbiome which prevents the establishment of foreign invaders, including pathogens.

Humans have a long history of co-evolution with our resident bacteria, and evidence suggests that our ancient hominid microbiota was more diverse and stable than that of modern humans (Adler et al., 2013; Moeller et al., 2014, 2016). Two dietary shifts, brought about by the development of agriculture (~7,500 years ago) and the Industrial Revolution (∼200 years ago) (Adler et al., 2013), significantly and rapidly increased the consumption of carbohydrates (particularly sucrose, in the case of the latter). These changes have perturbed the homeostasis of the oral microbiome and dental health, causing dental caries to become the most common chronic disease worldwide, affecting 60–90% of children and adults in industrialized countries (reviewed in Pitts et al., 2017). This review highlights therapeutic strategies, both contemporary and developing, that exploit the protective effects of the healthy oral flora in an effort to prevent dental caries.

## A Brief Overview of Dental Plaque Ecology

Typically, the earliest colonizers of the tooth surface are commensal streptococci, such as *Streptococcus mitis, Streptococcus sanguinis, Streptococcus gordonii*, and other closely related taxa. These species are the most avid binders of the naked, pellicle-coated tooth surface. Once these species have bound, they provide a more complex substrate to which other species can now bind. To help ensure their continued success, the majority of taxa within the mitis and sanguinis groups stanchly antagonize newcomers using the production of alkali, bacteriocins, and H_2_O_2_. In the absence of a carbohydrate-rich diet, these commensal streptococci tend to remain at high abundances in dental plaque. This dominance is strongly associated with good dental health. With frequent consumption of carbohydrates, particularly when concurrent with a lack of oral hygiene, increased bacterial production of a glucan matrix is favored, emeshing cells and preventing diffusion of metabolites. This allows for development of emergent properties of the dental plaque, such as acidic microenvironments resulting from carbohydrate fermentation. Typically, the saliva in the mouth has sufficient buffering capacity to neutralize the organic acids produced by bacterial metabolism, and repair acid-damaged enamel. However, the increased thickness and density of exopolysaccharide-rich plaque prevents both diffusion of saliva into the biofilm and diffusion of acids out of the biofilm. The commensal early colonizers are comparatively not well-adapted to acidic conditions, allowing for a further enrichment of acid-tolerant (aciduric) taxa such as *Streptococcus mutans, Veillonella* spp., and *Lactobacillus* spp. With progression of this positive feedback loop, the rate of net acid damage (demineralization) of the tooth enamel outpaces repair (remineralization), leading to clinical disease.

**FIGURE 1:**
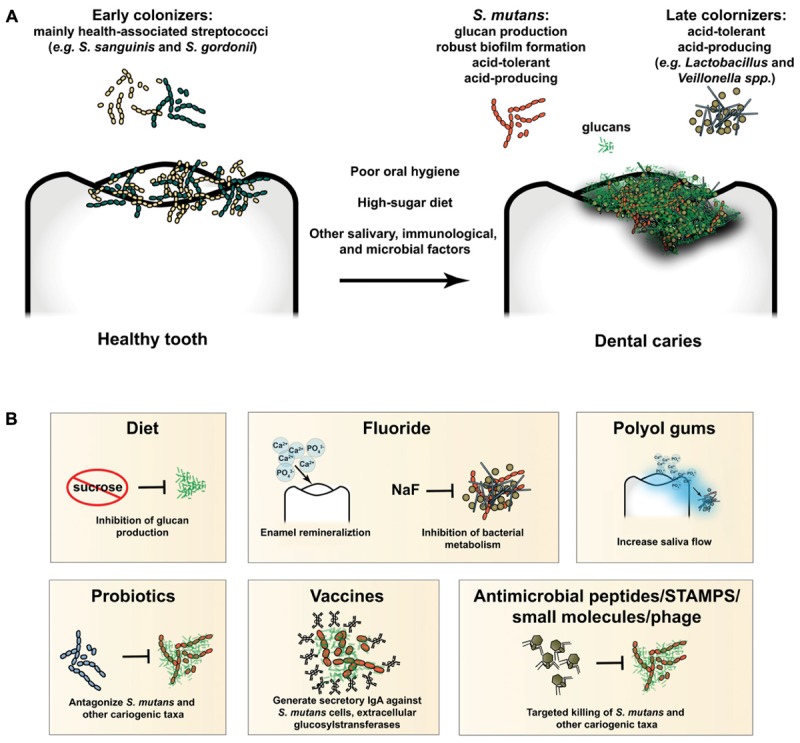
**(A)** Overview of caries pathogenesis. Early colonizers of the tooth are mainly health-associated streptococcal species, such as *S. sanguinis* and *S. gordonii*, as well as other closely related taxa. Poor oral hygiene, a high-sugar diet, and other salivary, immunological, and microbial factors lead to development of pathogenic biofilms (i.e., dysbiosis). *S. mutans* produces a glucan matrix, which leads to robust biofilm formation and colonization by taxa which could not have bound the tooth surface unassisted (late colonizers). Production of acid within the biofilm selects for increasingly acid-tolerant cariogenic organisms, such as *S. mutans, Lactobacillus spp.*, *Veillonella spp.*, and others. Unchecked, the process will destroy the protective enamel coating of the tooth and lead to clinical disease. **(B)** Preventative and therapeutic modalities in current use or development. A diet low in refined sugars, such as sucrose, will inhibit production of glucans and formation of cariogenic biofilms. Fluoride both promotes remineralization of tooth enamel and inhibits potentially cariogenic bacterial metabolism. Polyol gums increase saliva flow, delivering ions for tooth remineralization and promoting clearance of bacteria from the tooth surface. Probiotics antagonize and prevent establishment and outgrowth of pathogenic species, such as *S. mutans.* Immune priming via vaccination leads to elevated levels of secretory IgA, which binds target epitopes on *S. mutans* and other cariogenic targets, preventing binding and biofilm formation, and promoting clearance from the oral cavity. Antimicrobial peptides, STAMPS, small molecules, and phage promote targeted killing of specific cariogenic taxa, such as *S. mutans.*

With an arsenal of extracellular glucosyltransferases (Gtfs), *S. mutans* is particularly adept at producing a glucan matrix from sucrose, and therefore is considered a keystone species in caries pathogenesis (Bowen, 2016; Bowen et al., 2018). Competition between the early colonizers of the teeth and cariogenic species, particularly *S. mutans*, has been well-documented and acknowledged for decades (Marquis, 1995; Huang et al., 2018). Interested readers are directed to four excellent and recent reviews covering the above topics in more depth (Abranches et al., 2018; Bowen et al., 2018; Lamont et al., 2018; Redanz et al., 2018). This battleground over the ecological niche of the tooth surface represents a significant opportunity for intervention and subsequent prevention of caries. If the balance of power can be tipped in favor of health-associated organisms, it is possible that pathogenesis of caries can be halted. Figure 1 provides an overview of dental caries pathogenesis and the major preventative strategies discussed in this review.

## Current Control Measures: Diet and Fluoride

### Diet

Caries is not a classic infectious disease, but the consequence of an ecological shift. Indeed, pathogenic species are necessary, but not sufficient, to cause disease—a constant supply of carbohydrates is also required. In addition to dental caries, the carbohydrate-laden, highly processed modern Western diet has led to pandemics of obesity, type II diabetes, cardiovascular disease, as well as related metabolic disorders and cancers. As with caries, a large and growing body of evidence is linking these conditions to diet via microbial mediators (reviewed in Gilbert et al., 2018; Zmora et al., 2018). Education of the general public to the importance of diet, healthy dietary habits, and the significant association of diet, the microbiota, and health issues remains paramount. In addition to the dissemination of current dietary recommendations, improving the accessibility of healthy foods is a goal worthy of considerable attention. A return to a more primitive and unprocessed diet is likely to have significant health benefits by supporting the microbial profiles with which we have the proper evolutionary rapport to underpin a mutualistic relationship. This includes, of course, the microbial profiles on the tooth surface.

### Fluoride

Fluoride treatments, including fluoridated toothpaste and drinking water, have been used to combat dental caries for more than 50 years. Fluoride prevents and treats dental caries by promoting favorable remineralization of the tooth enamel while concomitantly impairing bacterial metabolism (Pitts et al., 2017). Although the efficacy of fluoride treatments is well-documented, clearly the current prevalence of the disease illustrates that fluoride alone is insufficient to prevent dental caries in many situations. Other, more stringent, antimicrobial agents are available for dental use (e.g., chlorhexidine and triclosan), but all are similarly broad-spectrum. As such, reengineering of a dysbiotic oral microbiome is likely to generate a more positive outcome than its total destruction. The development of approaches to specifically alter plaque composition and prevent outgrowth of cariogenic species, such as *S. mutans*, remains a highly attractive objective. These approaches fall into several broad categories, discussed below.

## Preventative Approaches in Development

### Prebiotics

Prebiotics are food or supplements which are administered to modulate the microbiome for the benefit of the host. Arginine has demonstrated success as a prebiotic to prevent dental caries (reviewed in Nascimento, 2018). Arginine can be broken down by commensal arginolytic species (e.g., *S. sanguinis* and *S. gordonii*) to generate ammonia, an alkaline molecule that buffers the organic acids in dental plaque. These reactions are performed by the arginine deiminase system (ADS). In addition to contributing to a more alkaline pH, the breakdown of arginine by the ADS also generates ATP, providing a bioenergetic advantage to the commensal streptococci (Bowen et al., 2018). Furthermore, arginine has been shown to inhibit the growth, pathogenic potential, and stress response mechanisms of *S. mutans*, thereby preventing caries pathogenesis through multiple mechanisms (Chakraborty and Burne, 2017). Higher cost, as well as controversy regarding the protective effects of arginine and the integrity of several clinical trials (Astvaldsdottir et al., 2016; Richards, 2017), have impeded dentifrices containing both fluoride and arginine from becoming widely available. As laboratory evidence for the protective effects of arginine continues to accumulate (Agnello et al., 2017; Huang et al., 2017; Zheng et al., 2017), more rigorous clinical trials could perhaps lead to widespread availability of arginine-containing commercial therapeutics. Recent studies have identified several other compounds; Met-Pro, succinic acid, beta-methyl-D-galactoside and *N*-acetyl-D-mannosamine as prebiotic candidates for caries prevention. These molecules were able to promote the dominance of health-associated organisms in a multispecies *in vitro* culture (Slomka et al., 2017, 2018). Whether these effects can be translated into *in vivo* studies remains to be investigated.

### Exploiting Oral Immunology

Caries is not immediately life-threatening, thus selective pressure on the host to thwart the condition is not terribly strong when compared to a disease like smallpox. On the other hand, teeth are a highly valued organ involved in obtaining and digesting food, self-defense, speech/communication, and even sexual attraction (Koussoulakou et al., 2009). It is likely no accident that moieties in the saliva provide binding sites and nourishment for specific species (i.e., the early colonizers), which are largely benign. Saliva flow, and the components of saliva have great influence over which taxa are able to persist in the mouth, and which are cleared (Marsh et al., 2016). Individuals with reduced salivary flow have a greatly increased prevalence of caries. Approaches to increase salivary flow are likely to assist in buffering acids, supplying antimicrobial peptides and antibodies, and preventing dysbiosis and caries from occurring. Chewing gums containing polyols, such as xylitol, provide salivary stimulus without fermentable carbohydrates. Furthermore, certain polyols, particularly erythritol (de Cock, 2018), also have antimicrobial activities, furthering their utility as a preventative modality (Makinen, 2010). Although auspicious, more rigorous research into the effects of these polyol molecules on overall systemic health is warranted; several other sugar substitutes have been recently shown to wreak havoc on the gut microbiota and promote disease (Suez et al., 2014; Rother et al., 2018).

Considering the adaptive arm of the immune system, levels of salivary IgA against immunogenic *S. mutans* epitopes, such as GTFs and glucan-binding proteins (GBPs), inversely correlate with colonization of *S. mutans* and caries prevalence (Taubman and Smith, 1993; Nogueira et al., 2005, 2012). Research investigating the feasibility of active or passive immunization against dental caries has been sporadic. Early investigations on the topic were excellently reviewed (Taubman and Nash, 2006). More recent studies have explored vaccination using a recombinant P1 adhesin antigen (Batista et al., 2017), a DNA-based vaccine against glucosyltransferases and surface proteins (Jiang et al., 2017), and a glycoconjugate vaccine based on rhamnan surface polysaccharides (St Michael et al., 2018). Unfortunately, past, present, and likely future, translational endeavors to move anti-caries vaccine research into clinical trials face significant regulatory and investment headwinds due to the fact that it is a non-life-threatening disease. There are currently no licensed vaccines to prevent dental caries.

### Probiotics

Aside from providing “food” for the oral microbiome to modulate ecology, ecology can also be directly altered by either selectively adding or removing particular species from the oral community. Attempted probiotic strategies to prevent caries either have sought to add health-associated taxa to bolster the capacity of the microbiome to resist dysbiosis, or to replace cariogenic strains with genetically modified mutants which are competitive, yet less pathogenic. A number of studies have explored the use of *Lactobacillus* and *Bifidobacterium*, traditionally the genera used in probiotic formulations for digestive health, in the prevention of dental caries. Despite some encouraging results (Lin et al., 2018), there is widespread skepticism concerning the use of these genera as anti-caries probiotics. Both *Lactobacillus* and *Bifidobacterium*, are acidogenic and aciduric, meaning that they may actually contribute directly to caries formation under the proper conditions, a fear supported by several studies (reviewed in Philip et al., 2018). In addition, most lactobacilli and bifidobacteria are residents of the gut, meaning they are not well-adapted for long-term persistence in the human mouth. They lack capabilities to bind to the salivary pellicle or even nascent dental plaques.

Species with a higher likelihood of outcompeting *S. mutans* are found already residing in the healthy oral cavity. Studies in other environments have illustrated that the best probiotics for preventing the growth of pathogens both occupy the same ecological niche as the pathogen, and produce compounds that directly antagonize the pathogen (Schlatter et al., 2017). *Streptococcus dentisani* and *Streptococcus* A12 are two recently described species which show particular promise as potential probiotics (Huang et al., 2016; Lopez-Lopez et al., 2017). Both species are active colonizers of the tooth surface, increase the pH of dental plaque through the arginolytic pathway, and inhibit the growth of mutans streptococci. In addition, *Streptococcus* A12 produces a challisin-like protease that disrupts pheromone signaling by *S. mutans*, inhibiting production of the bacteriocins which *S. mutans* utilizes to poison its competitors (Huang et al., 2016). Meanwhile, *S. dentisani* utilizes its own arsenal of bacteriocins to kill multiple cariogenic species (Lopez-Lopez et al., 2017). *Streptococcus salivarius* has also been examined in a probiotic context (Kurasz et al., 1986; Di Pierro et al., 2015). However, similar to lactobacilli and bifidobacteria, broad skepticism remains over the use of *S. salivarius* strains as dental plaque probiotics. *S. salivarius* is typically an inhabitant of the soft surfaces of the mouth and is thought to have limited ability to colonize the tooth surface and directly compete with *S. mutans in situ* (Philip et al., 2018).

The other major strategy used in probiotic approaches to prevent caries is displacement of native *S. mutans* strains with *S. mutans* strains engineered to have low pathogenicity. Two examples of this technique have been reported, utilizing *S. mutans* mutants deficient in intracellular polysaccharide metabolism (Tanzer et al., 1982) or lactic acid production (Hillman et al., 2007). Despite preliminary results supporting the potential of these strains as anti-caries probiotics, no further research or studies in humans have been reported. Overall, although newer candidates, such as *S. dentisani* and *Streptococcus* A12, provide encouragement, no formulations of probiotics have been tested in rigorous clinical trials and successfully received endorsement for the prevention of dental caries from a regulatory agency or professional organization (Gruner et al., 2016; Burne, 2018).

Recent studies have explored the capacity of the biosynthetic gene clusters (BGCs) encoded by the oral microbiome to produce compounds which modulate oral ecology (Donia et al., 2014; Aleti et al., 2018). Specific BGCs appear to be associated with health or disease states. Comparative statistical modeling illustrated that certain BGCs were inversely correlated with the abundance of cariogenic species, such as *S. mutans* and *Lactobacillus* spp. This indicates that the molecular products of these BGCs may be priority therapeutic leads and that the strains harboring these BGCs are prime probiotic candidates, inviting further investigation.

### Antimicrobial Peptides/STAMPS

As opposed to adding species to the community to prevent or alleviate dysbiosis, various approaches to remove problematic species, such as *S. mutans*, have been explored. Such targeted methods would presumably restore a healthy oral microbiome. Recent reports showed that the antimicrobial peptides ZXR-2 and CLP-4 efficiently killed *S. mutans* biofilms, however, specificity for *S. mutans* was not shown (Chen et al., 2017; Min et al., 2017). Specifically targeted antimicrobial peptides (STAMPS) are synthetic peptides consisting of a targeting domain to invoke specificity and a killing domain to exert antimicrobial action against the targeted species (Eckert et al., 2006b). C16G2 is a STAMP designed to specifically kill *S. mutans*, and several studies have shown that C16G2 is capable of targeted killing of *S. mutans* while leaving commensal streptococci unharmed. Furthermore, C16G2 was able to remodel the composition of a complex bacterial community, eliminating *S. mutans* and allowing for enrichment of organisms associated with dental health (Eckert et al., 2006a; Guo et al., 2015). C16G2 has proceeded to clinical trials in a number of formulations, the results of which will be of significant interest.

### Small Molecules

Several small molecules have been proposed as agents to prevent caries through disruption of *S. mutans* biofilms. The small molecule 3F1 selectively dispersed *S. mutans* biofilms and served to modestly reduce caries in rodent model, however, no changes in the oral microbiomes were reported aside from a moderate reduction in *S. mutans* as measured by CFUs (Garcia et al., 2017). A 2016 study identified a quinoxaline derivative capable of inhibiting the GtfC enzyme of *S. mutans*. The compound indeed successfully reduced the ability of *S. mutans* to form biofilms and reduced caries in a rat model (Ren et al., 2016). Although both of these approaches dispersed *S. mutans* biofilms, there was minimal effect on overall dental plaque ecology. It is likely that this will allow rapid reformation of the problematic community and require perpetual application of these therapeutics. A recent study utilized a drug repositioning approach to identify 126 compounds with activity against *S. mutans* (Saputo et al., 2018). How many of these leads are specific to *S. mutans* remains yet to be determined.

### Phage

Conceptually, bacteriophage is a very attractive approach to combat cariogenic pathogens, and one that has received relatively little attention. Although the few phages known to infect *S. mutans* were lytic, and completely eliminated viable counts from single-species biofilms, the phage demonstrated a highly stringent host specificity, which was considered a significant disadvantage, particularly in light of the high intra-species diversity exhibited by *S. mutans* (reviewed in Szafranski et al., 2017). No testing in multi-species communities or further studies have been reported to date.

## Conclusion and Perspectives

Because of its status as a key pathogenic species, much of the development of novel caries therapeutics has focused on *S. mutans*, specifically. *S. mutans* is certainly a justifiable target, associated with caries in the majority of cases and unparalleled in its ability to form insoluble glucans from sucrose. However, it is not the singular cause of the disease, and caries does occasionally occur without detectable *S. mutans* levels. Future research efforts would benefit from embracing this perspective and tempering expectations for approaches that fail to do so. It is clear that a dramatic reduction in the prevalence of dental caries through current modalities (such as fluoride and dietary modification) alone is unlikely to be realized. Fortunately, evolution has shaped territorial commensal taxa which antagonize cariogenic species. Exploitation of this relationship, whether by directly supporting the dominance of commensal taxa, or via targeted killing of their pathogenic competitors, is a promising course of therapeutic development. Although several of these approaches have produced encouraging results, properly controlled rigorous human studies are needed, the cost of which is likely to be a significant deterrent. Nevertheless, there is room for optimism, as it appears evolution may have already provided the best tools in the form of our commensal defenders and their natural products.

## Author Contributions

JB and AE reviewed literature and wrote the review.

## Conflict of Interest Statement

The authors declare that the research was conducted in the absence of any commercial or financial relationships that could be construed as a potential conflict of interest.
